# The association of long-term kidney function decline with mortality in patients with multiple myeloma: Single-center experience 

**DOI:** 10.5414/CNP104S15

**Published:** 2025-11-28

**Authors:** Rebeka Simić, Ana Rožič, Tadej Petreski, Nejc Pulko, Sebastjan Bevc

**Affiliations:** 1 Faculty of Medicine, University of Maribor,; 2 Department of Nephrology, Clinic for Internal Medicine, and; 3 Department of Hematology, Clinic for Internal Medicine, University Medical Center Maribor, Maribor, Slovenia; *These two authors have contributed equally to this work and share first authorship.

**Keywords:** multiple myeloma, kidney function decline, survival, mortality, eGFR slope

## Abstract

Introduction: Kidney dysfunction is a frequent complication of multiple myeloma (MM) and is associated with worse survival outcomes. Despite its prevalence, the prognostic value of long-term kidney function decline remains insufficiently explored. This study aimed to investigate the impact of the estimated glomerular filtration rate (eGFR) slope on survival in patients with MM. Materials and methods: A retrospective cohort study was conducted on 43 patients with MM treated at the University Medical Center Maribor between 2015 and 2020, with a minimum follow-up of 1 year. Kidney function was assessed quarterly using eGFR. Kaplan-Meier analysis and Cox regression were applied to evaluate the association between eGFR slope and overall survival. Results: The median baseline eGFR was 52.8 mL/min/1.73m^2^ (interquartile range: 35.2 – 78.1), with 39.5% of patients classified as having stage 3 or worse chronic kidney disease. We observed an association between faster annual eGFR slope decline and increased mortality (log-rank; p < 0.001). Cox regression confirmed eGFR slope as an independent predictor of mortality (hazard ratio = 1.121, 95% confidence interval: 1.069 – 1.174, p < 0.001). Additional prognostic factors included a lower platelet count. Conclusion: Kidney function decline is an independent prognostic factor in patients with MM. Regular monitoring and early nephrology intervention may help mitigate its impact. Future research should focus on targeted strategies to slow kidney function deterioration and improve patient outcomes.

## Introduction 

Multiple myeloma (MM) is a hematologic malignancy that arises as a clone of almost mature aberrant plasma cells. These malignant cells originate from post-germinal B lymphocytes and proliferate within the bone marrow microenvironment. The pathogenesis involves complex genetic and microenvironmental interactions that drive uncontrolled plasma cell growth and monoclonal immunoglobulin production [1]. MM is the second most common hematologic malignancy, accounting for ~ 10% of all hematologic cancers [1, 2]. The median age at diagnosis is 65 years, with fewer than 3% of cases occurring in patients younger than 40 years [3]. The median overall survival is ~ 6 years [4], and MM is 1.5 times more common in men than in women globally [5]. The typical clinical presentation of MM includes bone pain, pathological fractures, anemia, recurrent infections, hypercalcemia, kidney failure, and abnormal bleeding [3]. Although MM remains incurable, advancements in treatment have significantly improved patient outcomes. Many patients now achieve prolonged remission through a combination of chemotherapy and autologous hematopoietic stem cell transplantation (autoHSCT) [3]. The survival rate has more than doubled in recent decades due to novel chemotherapy regimens, monoclonal antibodies, bispecific antibodies, and chimeric antigen receptor (CAR) T-cell therapies. Initial therapy depends on eligibility for autoHSCT and comorbidity assessment (e.g., neuropathy, kidney injury) [5]. 

Kidney injury is a frequent and serious complication of MM, affecting 20 – 50% of patients. While kidney dysfunction can be reversed in ~ 50% of cases, many patients develop chronic kidney disease (CKD), with 2 – 12% progressing to end-stage kidney disease (ESKD) requiring kidney replacement therapy (KRT) [6]. The most common cause of progression to ESKD is light chain cast nephropathy, caused by the precipitation of monoclonal free light chains with uromodulin in the distal tubules. Additional contributing factors may include hyperuricemia, nonsteroidal anti-inflammatory drug (NSAID) use for bone pain, and dehydration [7]. However, less is known about chronic kidney function decline in MM. This study aimed to assess the long-term kidney function decline in MM patients and its prognostic implications. 

## Materials and methods 

This retrospective cohort study included patients aged ≥ 18 years diagnosed with MM and treated at the University Medical Center Maribor (UMCM) between January 1, 2015, and January 1, 2020. Follow-up data were collected until December 31, 2020. Patients with < 1 year of follow-up were excluded to ensure adequate observation time for kidney function assessment. Additionally, patients with myeloid leukemia, lymphoma, indolent plasmacytoma, amyloidosis, or those treated at other institutions were excluded. 

Clinical and laboratory parameters were recorded at diagnosis and monitored during follow-up. Kidney function was assessed via serum creatinine measurement and estimated glomerular filtration rate (eGFR) calculation using the 2009 CKD-EPI creatinine equation. Measurements and eGFR calculations were performed approximately every 3 months from inclusion until the end of observation, and each individual’s annual eGFR slope was calculated from all measurements. Patients were stratified into quartiles based on their annual eGFR decline so that all groups were equally represented. Baseline clinical parameters included blood count, serum albumin, β-2 microglobulin, lactate dehydrogenase (LDH), calcium (Ca) levels, and comorbidities such as diabetes mellitus (DM), arterial hypertension (AH), and dyslipidemia. 

Statistical analyses were conducted using IBM SPSS Statistics (version 26.0, SPSS Inc., Chicago, IL, USA). Data normality was assessed using the Shapiro-Wilk test. Normally distributed variables were presented as mean ± standard deviation (SD) and compared using Student’s t-test, while non-normally distributed variables were expressed as median and interquartile range (IQR) and analyzed using the Mann-Whitney U test. Categorical variables were analyzed using the χ^2^- or Fisher’s exact test. Survival analysis was performed using Kaplan-Meier survival curves, with significance determined by the log-rank test. A Cox proportional hazards model was used to determine independent prognostic factors for survival, adjusting for potential confounders such as age, comorbidities, and disease severity. Variables with a p-value of less than 0.05 in the univariate analysis were included in the multivariate model. Statistical significance was set at p < 0.05. 

The study was approved by the ethics committee at the UMCM (decision number UKC-MB-KME-42/22). 

## Results 

After accounting for exclusion criteria, 43 patients were analyzed, with a mean age of 67.1 ± 12.3 years, of whom 53.5% were female. The median eGFR at diagnosis was 52.8 mL/min/1.73m^2^ (IQR: 35.2 – 78.1), with 39.5% of patients having an eGFR below 60 mL/min/1.73m^2^. Several comorbidities were present, such as AH in 53.5%, DM in 23.3%, and dyslipidemia in 23.3%. Other demographic data are presented in Table 1. Biochemical results revealed mean hemoglobin (Hb) 98.9 ± 20.5 g/L, albumin 33.9 ± 8.0 g/L, paraprotein concentration 47.4 ± 25.2 g/L, median LDH 2.75 (IQR 0.70 – 4.80) μkat/L, and serum Ca 2.15 (IQR: 1.88 – 2.42) mmol/L. 

Except for 1 patient, all individuals received treatment for MM, with 65.1% undergoing first-line therapy with the bortezomib, thalidomide, and dexamethasone (VTD) regimen. Altogether, 49.1% received an autoHSCT. 

The median follow-up period was 984 ± 390 days, during which 55.8% of patients died. No patient had an acute kidney injury or progressed to ESKD requiring KRT. Patients with an eGFR below 60 mL/min/1.73m^2^ died more often than those with preserved kidney function (75.0 vs. 34.8%, p = 0.018). Additionally, deceased patients had lower platelet counts (175 vs. 252 × 10^9^/L, p = 0.023) and higher β-2 microglobulin levels (7.7 vs. 4.6 mg/L, p = 0.022). No significant differences were observed in performance status (PS), Hb, albumin, LDH, Ca, paraprotein concentration, or the presence of bone lesions. 

**Figure 1:**
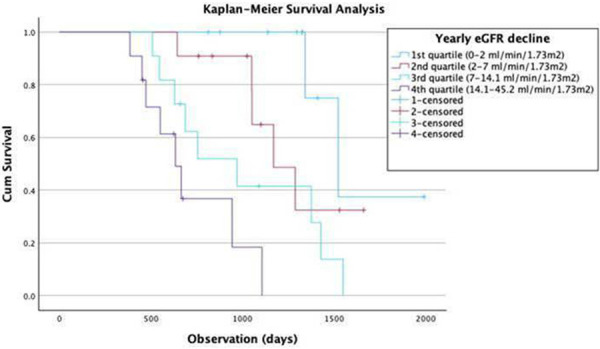
Kaplan-Meier survival analysis of yearly decline in estimated glomerular filtration rate in quartile groups.

The median annual eGFR decline was 8.38 mL/min/1.73m^2^ (IQR: –20.75 to 4.01). Kaplan-Meier survival analysis (Figure 1) demonstrated a strong correlation between yearly eGFR decline and survival. Patients in the first quartile (0 – 2 mL/min/1.73m^2^ per year) had the longest survival, while those in the fourth quartile (14.1 – 45.2 mL/min/1.73m^2^ per year) exhibited the shortest survival (log-rank test: χ^2^ = 23.916, p < 0.001). 

Cox regression (Table 2) confirmed that a steeper eGFR slope was independently associated with worse survival outcomes (hazard ratio = 1.121, 95% confidence interval: 1.068 – 1.174, p < 0.001). Additional significant predictors included a lower platelet count (p = 0.018). 

## Discussion 

Our study highlights the crucial role of kidney function decline, described as an annual drop of eGFR, in MM prognosis. A faster annual decline in eGFR was independently associated with increased mortality, even after adjusting for established prognostic factors. Patients with greater eGFR decline had significantly shorter survival times, emphasizing the need for regular kidney function monitoring in MM management. 

To our knowledge, no previous study has specifically examined the association between annual eGFR decline and mortality in patients with MM. Qian et al. [8] have shown that patients with MM and kidney impairment often develop CKD and that 40% of them receive nephrotoxic agents during treatment and follow-up. Furthermore, a recent study by Cesar et al. [9] has shown that anemia, hyperuricemia, proteinuria, and extramedullary plasmacytoma were associated with the development of CKD. 

Additionally, our study revealed a significant correlation between platelet count and survival, consistent with findings showing that thrombocytopenia is a risk factor for treatment-related mortality and disease progression in MM [10]. Blood cell disturbances are frequently observed in newly diagnosed patients with MM but can also result from chemotherapy treatment [11]. While our study did not find a significant impact of established risk factors (PS, Hb, albumin, LDH, Ca, paraprotein concentration, or bone lesions) on survival, previous studies have highlighted the prognostic value of Hb, albumin, and β-2 microglobulin [12]. Another study showed that higher values of β-2 microglobulin and LDH are correlated with inferior survival and associated with more significant tumor burden; however, β-2 microglobulin and LDH are also affected by renal failure and other comorbidities, which can confound the results and limit the utility of these biomarkers [13]. 

While our study provides valuable insights, certain limitations exist. As we only included patients treated at our hospital in a limited time frame, our sample for analysis was small. This is why we have also not analyzed genetic data or treatment response. Future research should focus on larger, prospective studies to validate these findings. It would also be valuable to explore which treatment approach for CKD has the most significant impact on kidney disease progression and complications in patients with MM. Current KDIGO guidelines for managing CKD recommend renin-angiotensin system inhibitors for managing CKD, such as angiotensin-converting enzyme inhibitors (ACEi) and angiotensin II receptor blockers (ARBs). Using sodium-glucose cotransporter 2 inhibitors (SGLT2i) is also advised [14]. No specific guidelines have yet been established for treating CKD in patients with MM [8]. 

In conclusion, preserving kidney function is critical for MM patients. The strong association between eGFR decline and survival highlights the need for aggressive kidney function monitoring and early intervention, maintaining fluid balance, and avoiding nephrotoxic medications as key components of MM treatment protocols. 

## Acknowledgment 

We want to acknowledge our colleagues from the hematology department for their clinical contribution in caring for patients with MM. 

## Authors’ contributions 

Conceptualization: T.P., S.B.; Methodology: T.P., N.P., S.B.; Data acquisition: R.S, A.R, T.P.; T.P.; Data analysis: T.P.; Writing – original draft preparation: R.S., A.R., T.P.; Writing – review and editing: T.P., N.P., S.B.; Supervision: S.B. 

All authors have read and agreed to the published version of the manuscript. 

## Funding 

This study received no funding. 

## Conflict of interest 

The authors have no conflict of interest to report. 

**Table 1.** Demographic characteristics of the study population.

Subject demographics	Percent
Sex (Female)	53.5
Smoking status:	
Never	55.2
Former	31.0
Current	13.8
ECOG performance status (0 – 6):	
0	14.0
1	23.3
2	39.5
3	18.6
4	4.7
Diabetes mellitus	23.3
Heart failure	11.6
Acute myocardial infarction	7.0
Atrial fibrillation	14.0
Arterial hypertension	53.5
Previous carcinoma	11.6
Dyslipidemia	23.3
CVI	9.3
Fatigue	72.1
Weight loss	46.5
Bone pain	79.1
Bone lesions	62.8
Osteoporosis	20.9
Bone Fractures	60.5
Immunoglobulins:	
IgA lambda	9.3
IgA kappa	7.0
IgG lambda	28.0
IgG kappa	37.2
Lambda light chains	9.3
Kappa light chains	7.0
Non-secretory	2.3
Dipstick proteinuria:	
None	30.2
1 – 4 = increasing in severity	34.9/14.0/14.0/7.0
Extramedullarity	14.3
Type of first-line chemotherapy:	
None	2.3
VTD	65.1
VMP	4.7
VCD	9.3
Dexamethasone	2.3
Thalidomide-dexamethasone	4.7
Bortezomib/dexamethasone	11.6

ECOG = eastern cooperative oncology group; CVI = cerebrovascular insult; Ig = immunoglobulin; VTD = bortezomib, thalidomide, dexamethasone; VMP = bortezomib, melphalan, prednisone; VCD = bortezomib, cyclophosphamide, dexamethasone.

**Table 2.** Cox regression analysis.

	B	SE	Wald	Sig	Exp(B)	Lower 95% CI	Upper 95% CI
Annual eGFR decline	0.114	0.024	22.868	**< 0.001**	1.121	1.069	1.174
Platelet count	–0.008	0.003	5.628	**0.019**	0.992	0.985	0.999
B2M	0.086	0.044	3.810	0.051	1.090	1.000	1.188

eGFR = estimated glomerular filtration rate; B2M = β-2 microglobulin.
